# Respiratory Syncytial Virus — United States, July 2012–June 2014

**Published:** 2014-12-05

**Authors:** Amber K. Haynes, Mila M. Prill, Marika K. Iwane, Susan I. Gerber

**Affiliations:** 1 Division of Viral Diseases, National Center for Immunization and Respiratory Diseases, CDC

Respiratory syncytial virus (RSV) causes lower respiratory infection among infants and young children worldwide (1). Annually in the United States, RSV infection has been associated with an estimated 57,527 hospitalizations and 2.1 million outpatient visits among children aged <5 years (2). In temperate climate zones, RSV generally circulates during the fall, winter, and spring (3). However, the exact timing and duration of RSV seasons vary by region and from year-to-year (4). Knowing the start of the RSV season in any given locality is important to health care providers and public health officials who use RSV seasonality data to guide diagnostic testing and the timing of RSV immunoprophylaxis for children at high risk for severe respiratory infection (5). To describe RSV seasonality (defined as onset, offset, peak, and duration) nationally, by U.S. Department of Health and Human Services (HHS) regions and for the state of Florida, CDC analyzes RSV laboratory detections reported to the National Respiratory and Enteric Virus Surveillance System (NREVSS). Florida is reported separately because it has an earlier season onset and longer season duration than the rest of the country (3). For 2012–13, the RSV season onset ranged from late October to late December, and season offset ranged from late December to late April, excluding Florida. For 2013–14, the RSV season onset ranged from late October to late January, and season offset from late January to early April, excluding Florida. Weekly updates of RSV national, regional, and state RSV trends are available from NREVSS at http://www.cdc.gov/surveillance/nrevss.

NREVSS records U.S. laboratory-based specimen data on RSV and other viral pathogens. Participating laboratories voluntarily report the aggregated numbers of tests performed and positive results each week (Sunday through Saturday). Season onset, offset, peak, and duration* are reported for each HHS region,† the state of Florida, and nationally, with and without Florida. This allows geographic variation in RSV activity to be described and accommodates the unusually early season onset and longer season duration observed in Florida compared with the rest of the United States (3). During July 7, 2012–June 29, 2013, approximately 93% of laboratories identified RSV by antigen detection methods (direct and indirect immunofluorescence antigen diagnostic tests). For this reason, and for consistency in reporting, only results from antigen detection methods are included in the analysis.

## 2012–13 Season

During July 7, 2012–June 2013, a total of 504 laboratories reported the results of at least 1 week of RSV testing by any testing method to NREVSS. For consistency, only results from antigen detection methods are included in the analysis. Antigen detection was used by 93% of participating laboratories during the 2012–13 season. CDC limited this analysis to 178 (35%) laboratories in 41 states that met the following criteria for inclusion: 1) reported RSV antigen testing results for ≥30 weeks during the 12-month NREVSS season and 2) averaged ≥10 antigen tests per week during the 52 weeks of the NREVSS season.§ Qualifying laboratories reported a total of 292,285 tests, of which 16% were positive for RSV. Nationally, RSV onset occurred the week ending October 27, 2012, and lasted 23 weeks until the week ending March 30, 2013 (Table). The proportion of specimens positive for RSV by antigen detection reached a season high of 25% during the week ending January 5, 2013. With Florida data excluded, the national onset occurred 2 weeks later (November 10, 2012), and the season duration decreased by 2 weeks compared with the national onset calculated with Florida data included. Onset for the 10 HHS regions (excluding Florida) ranged from late October to late December, and offset ranged from late December to late April (Figure). The season peak ranged from early December to early March, and the duration ranged from 6–23 weeks, with a median of 19 weeks (Table). Region 1 (Boston) had the shortest season, and Region 9 (San Francisco) had the longest. The season onset for Florida occurred the week ending July 21, 2012, and the season continued through the week ending January 26, 2013 (Table).

## 2013–14 Season

During July 6, 2013–June 28, 2014, a total of 408 (84%) laboratories identified RSV by antigen detection methods. The 2013–14 RSV season analysis is limited to 84 (21%) laboratories that met the inclusion criteria described previously. A total of 141,021 RSV antigen tests and 19,614 (12%) positive results were reported by eligible laboratories located in 33 states. Nationally, RSV onset occurred the week ending November 9, 2013, and lasted 21 weeks until the week ending March 29, 2014 (Table). The season peak occurred the week ending December 28, 2013. Excluding Florida, the national onset occurred 1 week later (November 16, 2013), and the season duration decreased by 1 week compared with the national onset, including Florida. Excluding Florida, the onset for the 10 HHS regions ranged from late October to late January, and offset ranged from late January to late April (Figure). Region 1 (Boston) and Region 2 (New York) had the shortest season, and Region 6 (Dallas) had the longest season. In Florida, the season onset occurred in the week ending July 6, 2013, and the season continued through the week ending January 25, 2014 (Table).

### Discussion

The national and regional RSV onsets for the 2013–14 season were similar to patterns previously reported (4). Florida’s season onset for the 2012–13 season occurred 3 weeks earlier than in the 2011–12 season and the 2013–14 season onset occurred 2 weeks before the 2012–13 season. Florida’s earlier onset has been well documented, as have differences in activity from year-to-year in the same geographic location (3). Social and demographic factors, population density, pollution, and climate each might influence national and regional RSV activity (3,6–8). Furthermore, RSV activity might vary between areas in the same region and areas in close proximity.

NREVSS surveillance data can be used to identify RSV activity and coordinate timing of immunoprophylaxis with palivizumab. Palivizumab is a monoclonal antibody recommended by the American Academy of Pediatrics to be administered to high-risk infants and young children likely to benefit from immunoprophylaxis based on gestational age, certain underlying medical conditions, and RSV seasonality (5).¶ NREVSS provides timely data on RSV trends at the national, regional, and state levels, which have correlated with RSV-associated hospitalizations in select regions (9). Consequently, physicians and public health professionals use NREVSS data to guide diagnostic testing to assess possible causes of regional outbreaks of respiratory infection. In a study using NREVSS data, a 5-year median onset and offset were calculated for individual laboratories and showed local RSV transmission did not always reflect regional trends (7). Surveillance data collected by state and local health departments as well as some children’s hospitals might more accurately describe RSV trends at a local level.

The findings in this report are subject to at least four limitations. First, reporting to NREVSS is voluntary and might be biased to over-represent more active reporters. Second, the percentage of laboratory tests that are positive each week reflects not only disease burden (i.e., number of cases per capita or severity of seasonal outbreaks) but also the volume of tests ordered. Third, NREVSS data cannot adequately quantify RSV activity in select locales because participation varies at the state and sub-state levels. Finally, periods of low RSV activity might not be captured by the NREVSS onset and offset definitions. Despite these limitations, NREVSS provides useful guidance to clinicians ordering diagnostic tests and planning to initiate immunoprophylaxis.

Weekly updates of national, regional, and state RSV trends are available from NREVSS at http://www.cdc.gov/surveillance/nrevss. Laboratories that wish to report data to NREVSS may register at https://wwwn.cdc.gov/nrevss/register/lab.aspx or contact NREVSS@cdc.gov for more information. Additional information regarding Florida RSV trends is available from the Florida Department of Health at http://www.doh.state.fl.us/disease_ctrl/epi/rsv/rsv.htm.

What is already known on this topic?

Respiratory syncytial virus (RSV) circulates in the United States from fall to spring, except in Florida, where circulation occurs from summer through spring. Knowing when the season has started and ended in any given locality is important for guiding diagnostic testing and the timing of prophylaxis for severe RSV infection. A network of laboratories report RSV testing results to the National Respiratory and Enteric Virus Surveillance System (NREVSS); annually, these data are summarized nationally and regionally.

What is added by this report?

During the 2012–13 season, RSV began circulating nationally in late October and ended in late March. Circulation peaked at 25% test positivity in early January. During the 2013–14 season, RSV began circulating nationally in early November and ended in late March. Circulation peaked at 24% in late December. These patterns in national RSV circulation were similar to those previously described. Onset and offset dates and season duration varied considerably among the regions and Florida.

What are the implications for public health practice?

Practitioners can use NREVSS data to determine which respiratory viruses are circulating in the United States and use the information to make decisions about the management of their patients with acute respiratory illness. Weekly updates of RSV national, regional, and state RSV trends are available from NREVSS at http://www.cdc.gov/surveillance/nrevss.

## Figures and Tables

**FIGURE:**
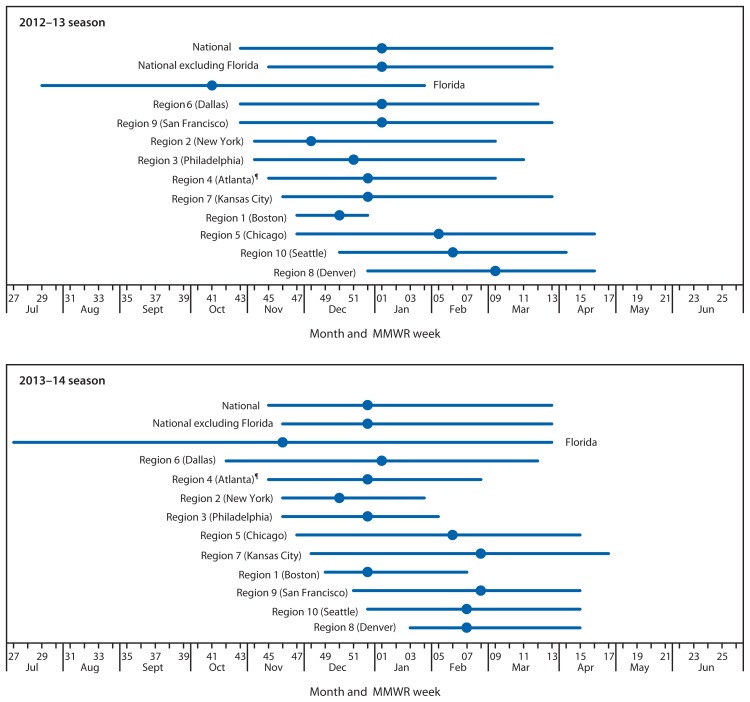
Respiratory syncytial virus season duration and peak, by U.S. Department of Health and Human Services Region,* and in Florida — National Respiratory and Enteric Virus Surveillance System, weeks ending July 7, 2012–June 29, 2013,^†^ and weeks ending July 6, 2013–June 28, 2014^§^ * Listed by region number and headquarters city. Region 1 (Boston): Connecticut, Maine, Massachusetts, New Hampshire, Rhode Island, and Vermont; Region 2 (New York): New Jersey and New York; Region 3 (Philadelphia): Delaware, District of Columbia, Maryland, Pennsylvania, Virginia, and West Virginia; Region 4 (Atlanta): Alabama, Florida, Georgia, Kentucky, Mississippi, North Carolina, South Carolina, and Tennessee; Region 5 (Chicago): Illinois, Indiana, Michigan, Minnesota, Ohio, and Wisconsin; Region 6 (Dallas): Arkansas, Louisiana, New Mexico, Oklahoma, and Texas; Region 7 (Kansas City): Iowa, Kansas, Missouri, and Nebraska; Region 8 (Denver): Colorado, Montana, North Dakota, South Dakota, Utah, and Wyoming; Region 9 (San Francisco): Arizona, California, Hawaii, and Nevada; Region 10 (Seattle): Alaska, Idaho, Oregon, and Washington. ^†^ District of Columbia, Idaho, Maine, Montana, Nevada, New Mexico, Oklahoma, Rhode Island, Vermont, and Wyoming did not have laboratories meeting the inclusion criteria for the 2012–13 season analysis. ^§^ District of Columbia, Alaska, Arizona, Delaware, Idaho, Illinois, Iowa, Kentucky, Maine, Montana, Nebraska, New Hampshire, New Mexico, Oklahoma, Rhode Island, Utah, Vermont, Wisconsin, and Wyoming did not have laboratories meeting the inclusion criteria for the 2013–14 season analysis. ^¶^ Excludes data from Florida.

**TABLE:** Summary of 2012–13 and 2013–14 respiratory syncytial virus seasons, by U.S. Departments of Health and Human Services (HHS) Region,* and in Florida — National Respiratory and Enteric Virus Surveillance System, June 2012–July 2014

	2012–13 season†					2013–14 season§				
		
HHS Region or state	No. of laboratories reporting	Onset week ending	Peak week ending	Offset week ending	Season duration (wks)	No. of laboratories reporting	Onset week ending	Peak week ending	Offset week ending	Season duration (wks)
National	174	10/27/2012	1/5/2013	3/30/2013	23	84	11/9/2013	12/28/2013	3/29/2014	21
National without Florida	156	11/10/2012	1/5/2013	3/30/2013	21	77	11/16/2013	12/28/2013	3/29/2014	20
Region 1 (Boston)	6	11/24/2012	12/15/2012	12/29/2012	6	4	12/7/2013	12/28/2013	2/15/2014	11
Region 2 (New York)	15	11/3/2012	12/1/2012	3/2/2013	18	6	11/16/2013	12/14/2013	1/25/2014	11
Region 3 (Philadelphia)	19	11/3/2012	12/22/2012	3/16/2013	20	6	11/16/2013	12/28/2013	2/1/2014	12
Region 4 (Atlanta)¶	24	11/10/2012	1/19/2013	3/2/2013	17	19	11/9/2013	12/28/2013	2/22/2014	16
Region 5 (Chicago)	24	11/24/2012	2/2/2013	4/20/2013	22	11	11/23/2013	2/8/2014	4/12/2014	21
Region 6 (Dallas)	24	10/27/2012	1/5/2013	3/23/2013	22	12	10/19/2013	1/4/2014	3/22/2014	23
Region 7 (Kansas City)	11	11/17/2012	12/29/2012	3/30/2013	20	3	11/30/2013	2/22/2014	4/26/2014	22
Region 8 (Denver)	7	12/29/2012	3/2/2013	4/20/2013	17	4	1/18/2014	2/15/2014	4/12/2014	13
Region 9 (San Francisco)	18	10/27/2012	1/5/2013	3/30/2013	23	9	12/21/2013	2/22/2014	4/12/2014	17
Region 10 (Seattle)	8	12/15/2012	2/9/2013	4/6/2013	17	3	12/28/2013	2/15/2014	4/12/2014	16
Florida	18	7/21/2012	10/13/2012	1/26/2013	28	7	7/6/2013	11/16/2013	1/25/2014	30

* Listed by region number and headquarters city. Region 1 (Boston): Connecticut, Maine, Massachusetts, New Hampshire, Rhode Island, and Vermont; Region 2 (New York): New Jersey and New York; Region 3 (Philadelphia): Delaware, District of Columbia, Maryland, Pennsylvania, Virginia, and West Virginia; Region 4 (Atlanta): Alabama, Florida, Georgia, Kentucky, Mississippi, North Carolina, South Carolina, and Tennessee; Region 5 (Chicago): Illinois, Indiana, Michigan, Minnesota, Ohio, and Wisconsin; Region 6 (Dallas): Arkansas, Louisiana, New Mexico, Oklahoma, and Texas; Region 7 (Kansas City): Iowa, Kansas, Missouri, and Nebraska; Region 8 (Denver): Colorado, Montana, North Dakota, South Dakota, Utah, and Wyoming; Region 9 (San Francisco): Arizona, California, Hawaii, and Nevada; Region 10 (Seattle): Alaska, Idaho, Oregon, and Washington.

† District of Columbia, Idaho, Maine, Montana, Nevada, New Mexico, Oklahoma, Rhode Island, Vermont, and Wyoming did not have laboratories meeting the inclusion criteria for the 2012–13 season analysis.

§ District of Columbia, Alaska, Arizona, Delaware, Idaho, Illinois, Iowa, Kentucky, Maine, Montana, Nebraska, New Hampshire, New Mexico, Oklahoma, Rhode Island, Utah, Vermont, Wisconsin, and Wyoming did not have laboratories meeting the inclusion criteria for the 2013–14 season analysis.

¶ Excludes data from Florida.

## Footnotes

* In NREVSS, the onset week in an area (national, region, or state) is defined as the first of 2 consecutive weeks when the weekly percentage of all specimens testing positive for RSV antigen in all reporting laboratories in the area is ≥10%. The offset is the end of the last 2 consecutive weeks when the weekly percentage positive exceeds 10%. The peak is the week when the percentage of positive RSV antigen tests is the highest. The season duration is comprised of the onset week, the offset week, and the weeks between.

† Listed by region number and headquarters city. Region 1 (Boston): Connecticut, Maine, Massachusetts, New Hampshire, Rhode Island, and Vermont; Region 2 (New York): New Jersey and New York; Region 3 (Philadelphia): Delaware, District of Columbia, Maryland, Pennsylvania, Virginia, and West Virginia; Region 4 (Atlanta): Alabama, Florida, Georgia, Kentucky, Mississippi, North Carolina, South Carolina, and Tennessee; Region 5 (Chicago): Illinois, Indiana, Michigan, Minnesota, Ohio, and Wisconsin; Region 6 (Dallas): Arkansas, Louisiana, New Mexico, Oklahoma, and Texas; Region 7 (Kansas City): Iowa, Kansas, Missouri, and Nebraska; Region 8 (Denver): Colorado, Montana, North Dakota, South Dakota, Utah, and Wyoming; Region 9 (San Francisco): Arizona, California, Hawaii, and Nevada; Region 10 (Seattle): Alaska, Idaho, Oregon, and Washington. The District of Columbia, Idaho, Maine, Montana, Nevada, New Mexico, Oklahoma, Rhode Island, Vermont, and Wyoming did not have laboratories meeting the inclusion criteria for the 2012–13 season analysis. The District of Columbia, Alaska, Arizona, Delaware, Idaho, Illinois, Iowa, Kentucky, Maine, Montana, Nebraska, New Hampshire, New Mexico, Oklahoma, Rhode Island, Utah, Vermont, Wisconsin, and Wyoming did not have laboratories meeting the inclusion criteria for the 2013–14 season analysis.

§ The 12 months included in a particular reporting season that runs from July through June.

¶ CDC does not make recommendations regarding the administration of RSV immunoprophylaxis.
