# Errata

**Published:** 2014-12-12

## Vol. 63, No. 48

In the report, “Respiratory Syncytial Virus — United States, July 2012–June 2014,” two errors occurred. In the figure on page 1135, the line depicting respiratory syncytial virus season duration in Florida should not extend beyond January 25 for the 2013–14 season. Also, in the summary box on page 1136, under the heading “What is already known on this topic?,” the first sentence should read, “Respiratory syncytial virus (RSV) circulates in the United States from fall to spring, except in Florida, where circulation **can occur** from summer **into winter**.

